# Changes in Affective Control Covary with Changes in Mental Health Difficulties Following Affective Control Training (AffeCT) in Adolescents

**DOI:** 10.1017/S0033291723002167

**Published:** 2023-08-23

**Authors:** Susanne Schweizer, Jovita T. Leung, William Trender, Rogier Kievit, Adam Hampshire, Sarah-Jayne Blakemore

**Affiliations:** 1 School of Psychology, University of New South Wales; 2 Department of Psychology, University of Cambridge; 4 The Computational, Cognitive and Clinical Neuroimaging Laboratory, Imperial College London; 5 Cognitive Neuroscience Department, Donders Institute for Brain, Cognition, and Behavior, Radboud University Medical Center

## Abstract

**Background:**

Everyday affective fluctuations are more extreme and more frequent in adolescence compared to any other time in development. Successful regulation of these affective experiences is important for good mental health and has been proposed to depend on affective control. The present study examined whether improving affective control through a computerized affective control training app (AffeCT) would benefit adolescent mental health.

**Methods:**

One-hundred and ninety-nine participants (11-19 years) were assigned to complete two weeks of AffeCT or placebo training on an app. Affective control (i.e., affective inhibition, affective updating and affective shifting), mental health and emotion regulation were assessed at pre- and post-training. Mental health and emotion regulation were assessed again at one month and twelve months later.

**Results:**

Compared with the placebo group, the AffeCT group showed significantly greater improvements in affective control on the trained measure. AffeCT did not, relative to placebo, lead to better performance on untrained measures of affective control. Pre- to post-training change in affective control covaried with pre- to post-training change in mental health problems in the AffeCT but not the placebo group. These mental health benefits of AffeCT were only observed immediately following training and did not extend to one month or year post-training.

**Conclusion:**

In conclusion, the study provides preliminary evidence that affective control training may confer short-term preventative benefits for adolescent mental health.

Negative and positive affective states are more labile during adolescence (10–24 years; Sawyer, Azzopardi, Wickremarathne, & Patton, 2018) compared with in adulthood (Bailen, Green, & Thompson, 2019; Green et al., 2021; Griffith, Clark, Haraden, Young, & Hankin, 2021). Dysregulated affect is a core characteristic of common mental health disorders, including depressive and anxiety disorders. Regulation of affective states depends on the deployment of situationally appropriate emotion regulation strategies (Silvers & Guassi Moreira, 2019). The ability to select adaptive regulatory strategies depending on contextual demands (e.g., engaging in action-focused strategies is not adaptive when the situation is not changeable) relies on affective control. Affective control refers to the application of cognitive control in affective contexts, that is, the capacity to inhibit affective information that is in conflict with current goals, while attending and responding to goal relevant environmental and internal inputs (Schweizer et al., 2020). Affective control is still developing during adolescence (Aïte et al., 2018) and might constitute a promising target for prevention and early intervention (S. Schweizer, Gotlib, et al., 2020). Here, we explore the preventative potential of affective control training for reducing mental health symptoms in adolescents.

Computerized affective control training has been shown to improve emotion regulation capacity and mood in healthy and clinical adult samples (Krause-Utz et al., 2020; Lotfi et al., 2021; Pan, Hoid, Gu, & Li, 2020; Pan, Hoid, Wang, Jia, & Li, 2020; Veloso & Ty, 2021). Neuroimaging evidence has shown that these training-related affective benefits are associated with increased recruitment of the cognitive control network, particularly the ventrolateral node (S. Schweizer, Grahn, Hampshire, Mobbs, & Dalgleish, 2013). This region, the inferior frontal gyrus of the ventrolateral prefrontal cortex, is recruited more frequently during affective control when compared with neutral (cool) cognitive control (S. Schweizer, Satpute, et al., 2019). The cognitive control network develops throughout adolescence, with the inferior frontal gyrus being one of the structures to show the latest structural maturation (Dong, Margulies, Zuo, & Holmes, 2021). Training affective control during adolescence might be especially advantageous as brain development is experience-dependent (Frankenhuis & Walasek, 2020). Training could offer additional opportunities to apply affective control during a stage when this capacity and its underlying neural substrates are still developing.

Before discussing the affective control training (AffeCT) used in the current study, it should be noted that ‘cool’ cognitive control training, where cognitive control is trained on valence-neutral tasks (including, digits, letters and other valence neutral stimuli), has also been shown to benefit mental health in both adolescents and adults. For example, working memory training (with no affective component) was shown to reduce the onset of depressive symptoms in a school-based sample of adolescents (Beloe & Derakshan, 2020). Research with adults has shown that cognitive control training improves both negative affect and affect regulation (Calkins, McMorran, Siegle, & Otto, 2015; Hoorelbeke, Koster, Demeyer, Loeys, & Vanderhasselt, 2016; Hoorelbeke, Koster, Vanderhasselt, Callewaert, & Demeyer, 2015; Onraedt & Koster, 2014; Owens, Koster, & Derakshan, 2013; Siegle, Thompson, Carter, Steinhauer, & Thase, 2007). Considering the efficacy of cool cognitive training, is there a need to train affective control? Preliminary evidence suggests so: Affective control has been shown to be uniquely associated with clinical endpoints that are central to the onset, relapse and maintenance of depression in young people, such as rumination (Hilt, Leitzke, & Pollak, 2014, 2017; Hilt & Pollak, 2013). Training affective control, therefore, may confer benefits to mental health over and above those observed for cool cognitive control interventions.

In the present study we tested AffeCT (Susanne Schweizer et al., 2022), which trains affective control on an affective working memory task. The training requires participants to continuously update affective information (words and faces) in working memory. AffeCT includes three tasks, single modality (separate auditory and visuospatial) *n*-back tasks as well as a dual *n*-back task in which the two modalities have to be attended to simultaneously. The *n*-back paradigm requires individuals to indicate whether the current stimuli they are seeing and/or hearing are the same as those presented a specified number of trials ago (i.e., *n*-back). Together, these AffeCT tasks train engagement with task-relevant affective information (auditory modality) and disengagement from task-irrelevant affective properties (visuospatial modality), or both.

To evaluate the efficacy of affective control training in improving our clinical outcomes of interest (mental health, affect and emotion regulation), we adopted the ‘Science of Behaviour Change framework’ experimental medicine approach (Nielsen et al., 2018). The premise of the framework is to evaluate interventions by identifying a target mechanism, in this case affective control, and investigate whether change in the target mechanism drives change in the clinical outcome of interest. To this end, a reliable assay of the target mechanism is required. Affective control here is operationalised as including three different facets: affective inhibition, affective shifting and affective working memory (S. Schweizer, Gotlib, et al., 2020). Any training that successfully improves affective control might lead to improvements in any, or all, of the facets of affective control. The present study therefore included a multifaceted assessment of affective control, including the affective backward digit span task (S. Schweizer, Leung, et al., 2019) as a measure of affective working memory updating, the affective Stroop task as a measure of affective inhibition (Preston & Stansfield, 2008), and the affective card sorting task (S. Schweizer, Parker, Leung, Griffin, & Blakemore, 2020) as a measure of affective shifting. In addition to examining the effect of training on each facet separately, we examined the structure of affective control in this sample to extract a meaningful assay of affective control. This index of affective control is essential to test whether any improvements in the clinical outcomes of interest vary as a function of changes in the index of affective control. As per protocol (S. Schweizer, Leung, et al., 2019), we predicted that performance on these affective control tasks would dissociate into separable affective control and cognitive control factors. This two-factor model was compared to a single factor model, which would indicate that there is no difference between affective control applied in affective or neutral contexts.

Pre- to post-training changes on these indices of affective control were compared between the AffeCT group and a group undergoing placebo training (Placebo). The Placebo group received the same narrative regarding the potential benefits of training on mental well-being and affect regulation. Placebo training was included to control for any effects of engaging repeatedly in a computerized cognitive training activity purported to benefit well-being and emotion regulation.

This design allowed us to test the following pre-registered hypotheses(S. Schweizer, Leung, et al., 2019): *Affective control training hypothesis (H1):* affective control can be improved in adolescents. To examine the first hypothesis, we compared pre- to post-training affective *n*-back performance across the two training groups. *Affective control facets hypothesis (H2):* Compared to Placebo, AffeCT would lead to greater improvements in all facets of affective control. To investigate this hypothesis, changes in performance on the affective transfer tasks were compared between the training groups. *Age-related change hypothesis (H3):* Training-related changes in affective control would vary as a function of age. *Mental health hypothesis (H4):* Improved affective control from pre- to post-training would be associated with fewer self-reported mental health problems, emotion regulation difficulties and self-control ability.

## Methods

### Participants

Two-hundred and forty-two participants aged 11-19 years old were recruited from eleven schools from Greater London, as well as through advertisements at University College London and the University of New South Wales. 43 participants were excluded due to technical issues or not meeting inclusion criteria, for details see the participant inclusion flowchart Figure S1. The final sample included 199 participants (159 female, mean age = 14.32 years, SD = 2.31 years, Table 1 for participant characteristics) who were randomized to one of the two training groups: Affective Control Training (AffeCT; *n* = 101), or the Placebo group (Placebo; *n* = 98). Training allocation was based on a computer-generated condition assignment (using Sealed Envelope simple randomisation service) stratified by age (young adolescents 11–14 years and mid-late adolescents 15–19 years). Following randomisation, the groups were matched on all baseline characteristics including age, gender, socioeconomic status, ethnicity, fluid intelligence, mental health, emotion regulation and self-control.

### Ethical standards

The authors assert that all procedures contributing to this work comply with the ethical standards of the Helsinki Declaration of 1975, as revised in 2008. The study was approved by the University College London [Ref: 12753/002] and UNSW Research Ethics Committees [HC3231].

### Testing procedure

Informed consent was obtained from parents if the participant was under 18 years and from participants aged 18 or over; participants under 18 also provided informed assent. The pre- and post-training sessions each lasted 1.5 hours and took place at the school or in the research lab in groups between 2 to 42 participants (with 1-4 researchers in each session). During the pre- and post-training sessions, participants completed a range of cognitive tasks and questionnaires. The mental health, emotion regulation and self-control questionnaires were administered to participants again online at two follow-up assessments, one month and twelve months after the post-training assessment. Researchers involved in the post-training sessions were blinded to training-group allocation. Participants were compensated £10 for each pre- and post-training session, £2/training day (£5 if they completed more than one training session in a single day), and £5 for each follow-up questionnaire.

### Training procedure

Participants were asked to complete 14 days of online training on their own device in a quiet space within a 4-week period. On the first three day both groups completed a different version (A-C) of the training tasks. From day four onwards, participants freely choose any version of the training. Both groups were told to spend as much time as possible training on version C due to its benefits to attention, memory, and emotion regulation. However, to maximise training engagement participants had the option to continue using the other versions. A full training session took between 20-30 mins, depending on the performance levels achieved. Training could be ended after 10 mins. Training sessions under 10 mins were not considered a full training session and not included in the analyses. Participants received a daily training reminder at 8am. Those who did not complete the training by 5pm received an additional reminder.

### Tasks and measures

#### Affective control training task

**Figure 1:**
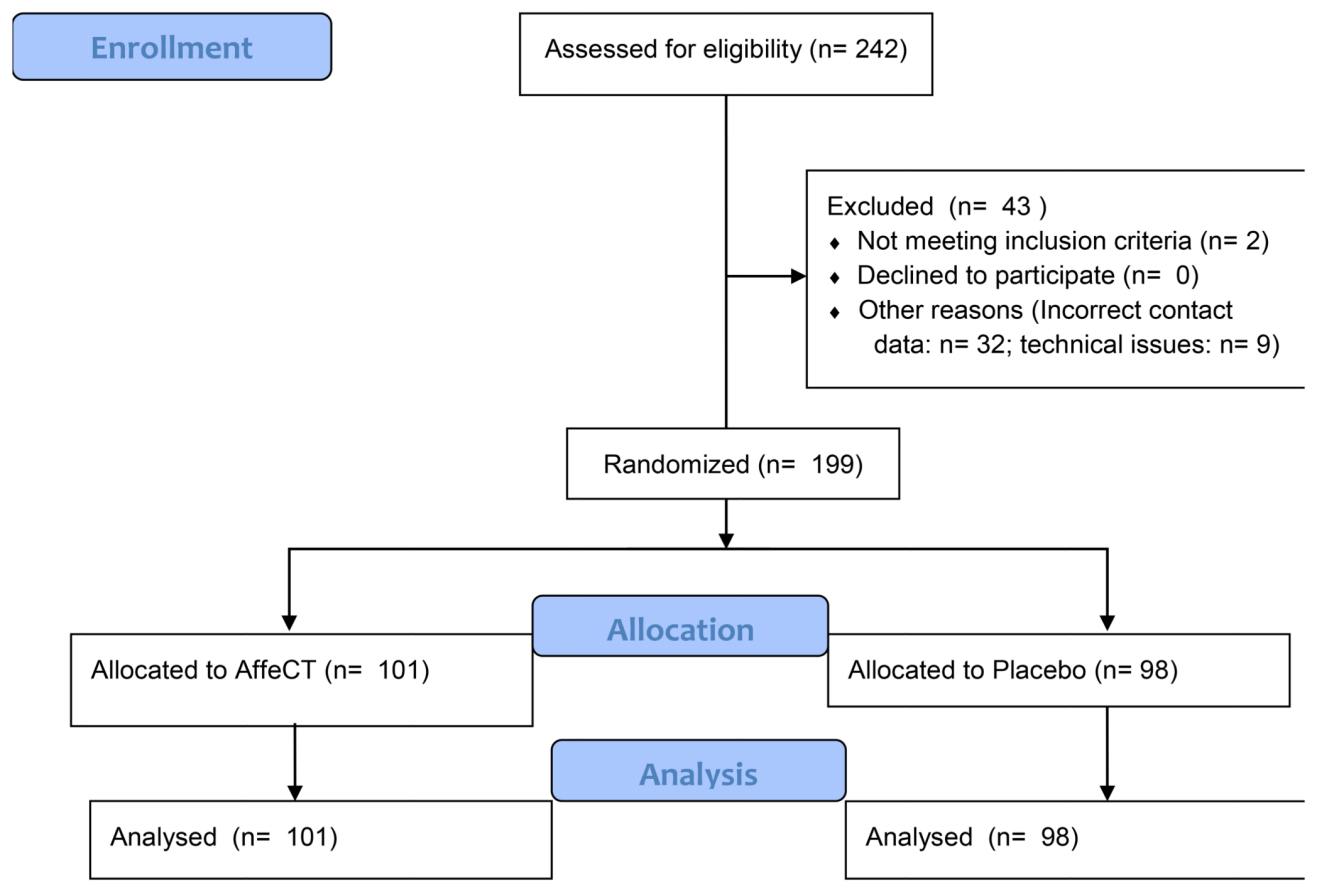
AffeCT Tasks Including a Visuospatial (A), Auditory (B) and Dual (C) n-back Task *Note*. The figure depicts sample trials for each of the three training tasks: A) visuospatial *n*-back, B) auditory *n*–back, and C) dual *n*-back task. Trials depicted with a light blue background require a “No Match” response. Yellow backgrounds indicate “Match” (i.e., target) trials. The example block in Figure 1 is depicted for *n* = 1.

AffeCT consisted of three versions of the *n*-back task (Figure 1): a visuospatial *n*-back (A), an auditory *n*-back (B) and a dual (including the auditory and visuospatial modalities) *n*-back (C). Across all three versions of the task, stimuli were presented on a 4 x 4 grid and/or over headphones. Participants had to respond via a “Match” or “No match” button press to indicate if the stimuli matched the corresponding stimuli presented *n* trials back (for details see Supplementary Materials).

#### Placebo training task

The Placebo task required participants to indicate via button press (“Match”, “No Match”) whether two panels displayed exactly the same stimuli in the same positions on a grid. There were three versions of the task that differed in the type of stimuli they included, namely: shapes, faces and word. The faces and words versions include the same stimuli as AffeCT. The initial trial included 5 items per panel, the number of items per panel subsequently increased with participants’ performance.

#### Affective control tasks

To assess the different facets of affective control three measures of affective inhibition, updating and shifting were included. These measures were administered before and immediately after training.

##### Inhibition

The affective Stroop task was used to assess inhibition of affective interference (Preston & Stansfield, 2008). Pictures of faces were presented to the participants with words superimposed on the image. Then, participants were asked to indicate whether the adjectives were happy or sad. Feedback was provided after each trial with a red or green boarder around the image for 200 ms to indicate an incorrect or correct response. The task was self-paced and there were 96 trials in total. Trials were considered inaccurate if no response was detected after 4 s. The performance of the task was operationalised as task accuracy (i.e., percentage trials correct) and reaction time was recorded.

##### Updating

The affective backward digit-span task was used to assess updating. Participants were presented with a series of digits (1500 ms) displayed over negative or neutral background images. Participants were then asked to recall the digits in reverse order. The task started at two digits, with each span level presented twice. To progress to the next span level, participants had to get at least one out of the two trials correct. The task was terminated if both trials were incorrect. The performance of the task was operationalised as the highest span level achieved in the negative and neutral condition.

##### Set-shifting

The affective set-shifting task, which was adapted from the Madrid Card Sorting Task (Schweizer, Parker, et al., 2020), was used to assess individual’s capacity to switch between task demands. Participants were dealt with a card and were asked to assign it to one of the four decks according to the three possible sorting rules: 1) card colour, 2) number of items on the card, and 3) shapes (for neutral condition) or emotional expressions (for affective condition). There were 96 trials in total and the sorting rule changed randomly after 6 to 9 trials. Participants had to respond within 30 s or the trial would be recorded as an error. Performance of the task was operationalised as random errors, which refers to errors that occur on any trial in the series from the third trial onwards (as the first two trials were needed to establish the correct sorting rule).

#### Mental health, emotion and self-regulation

The 25-item Strengths and Difficulties Questionnaire (SDQ; Goodman, 1997) was used to assess mental health difficulties. The scale has been developed for use in children and adolescents (Goodman, 1997). Participants indicated the extent to which the statements were true of them from “0”*Not true* to “2”*Certainly true*. Internal consistency in the current sample was acceptable, *ωT* = 0.81.

The Difficulties in Emotion Regulation Scale (DERS; Gratz & Roemer, 2004) assessed emotion regulation. The 36-item scale measures a range of affective processes and has been shown to be valid for use in adolescents (Charak et al., 2019). Participants indicated how often from “0”*Almost never* to “4” *Almost always* they experienced difficulties with different facets of emotion regulation. Internal consistency in the current sample was good, *ωT* = 0.93.

Self-regulation was assessed using the Brief Version of the Self-Control Scale (Tangney et al., 2004), which has been successfully used with adolescents (Duckworth, Kim, & Tsukayama, 2013). The scale includes 13 statements and participants had to indicate, on a 5-point scale from “0”*Not at all* to “4” *Very much*, how well each statement described them. Internal consistency in the current sample was acceptable, *ωT* = 0.89.

#### Fluid intelligence

At pre-training we additionally administered the 12-item version of the Raven’s Advanced Progressive Matrices to compare the groups on pre-training differences in fluid intelligence (Raven et al., 1988). Participants were instructed to complete the task as quickly as possible. The measure has good psychometric properties (Raven, 2000).

### Statistical analysis

To investigate gain on the affective control training task, our protocol specified that this would be analysed investigating dprime (*d’*) scores as performance index. However, the inclusion of a no-match button in the task design meant that hit rates were at ceiling, rendering the *d*’prime scores non-informative. We therefore opted to examine the maximum level of *n*-back achieved instead, as is conventional for *n*-back training studies (Soveri, Antfolk, Karlsson, Salo, & Laine, 2017).

The protocol specified a multivariate mixed effects model for the analyse of the three facets. However, as the primary outcomes varied across tasks (accuracy for the affective digit span task and reaction time for the affective Stroop and Card Sorting tasks) the facets were analysed in individual mixed effects models. The results were Bonferroni-corrected (.05/3) for three separate comparisons and therefore the statistical threshold was α =.017. All analyses were conducted in R, version 4.1.0 (R Core Team, 2013).

## Results

### Baseline affective control

In line with our pre-registration, we examined the structure of affective control at baseline. Specifically, we predicted affective control and cognitive control to be correlated but separate factors. The predicted two-factor model including performance on the transfer measures of shifting, inhibition and updating did not converge, even after scaling the reaction time data. Removing the latent congruency factor from the latent affective control factor allowed the model to converge but showed a very poor fit (*X^2^*(96)=800.81; CFI=.37; TLI=.21; RMSEA=.21; SRMR=.20; AIC=567.38). We therefore examined the structure for accuracy and reaction time separately. The two-factor structure provided a good fit for the reaction time data. However, the fit was not significantly better when compared to a model including a single cognitive control factor, Δ*X^2^*(3)=4.27; *p*=.234. For accuracy, the two-factor showed borderline acceptable fit (Table S1) and outperformed the poorly fitting one-factor model Δ*X*(3)=41.32; *p*<.001. The latent affective control factors (from the two-factor models) for accuracy and reaction time were retained to examine hypothesis 4, as per protocol.

### Training characteristics and results

There were no significant differences (see supplementary materials) in training adherence with the exception of average number of sessions completed, which was significantly higher in the Placebo (*M*=12.08, *SD*=13.39) compared to the AffeCT group (*M*=7.19, *SD*=9.12), *t*(187)= 2.90, *p*=.004. Number of training sessions completed was therefore included as a covariate.

In line with our *affective control training hypothesis*, which predicted that the AffeCT group would perform better on the affective *n*-back task following training, there was a Training group x Time interaction, *b*=0.48, *SE*=0.13, *t*=3.70, *p*<.001. The interaction remained significant when controlling for number of sessions trained, *b*=0.47, *SE*=0.13, *t*=3.55, *p*<.001 (for full model statistics see Table S2).

### Effects of training on the three components of affective control

We found no support for our second *affective control facets* hypothesis: AffeCT did not lead to greater gains in affective inhibition, shifting or updating. As indicated by non-significant interactions between the effects of training group (AffeCT vs. Placebo) and time (Pre vs. Post-training) reported in Table 2.

### Age-related differences in affective control training

In line with our third *age-related change* hypothesis, there were age-related differences in training gains on the affective *n*-back task. That is, age group moderated the significant group by time interaction reported in H1, *b*=0.24, *SE*=0.07, *t*=3.56, *p*<.001 (for the full model estimates see Table S4). This effect was reduced but remained significant when correcting for number of training sessions completed, *β*=0.06, *SE*=0.02, *t*=3.50, *p*<.001. Analyses of the estimated marginal means trends revealed a significant age by time interaction effect in the AffeCT group, *b*=–0.17, *SE*=0.08, *t*=–2.30, *p*=.022, but not in the Placebo group, *b*=0.08, *SE*=0.07, *t*=1.23, *p*=.219. In the AffeCT group increasing age showed a small, non-significant association with improvements on the affective *n*-back task, *r*=.22, *p*=.069.

### The effect of training on mental health, emotion regulation and self-control

Applying a multi-group latent growth curve model showed that AffeCT, but not Placebo, training led to significant covariance of change in affective control index identified at baseline in the reaction time and accuracy models and mental health difficulties (Table S5). The formal comparison of the free model to a model with constrained variance of the latent variables was significant for both affective control indices: reaction time: *X_Diff_^2^*=11.07, *p*=.004; accuracy: *X_Diff_^2^*=10.04, *p*=.007.

Extracting the indices of change from the latent growth curve model revealed that post-training mental health difficulties in the AffeCT group were negatively associated with change in affective control, (reaction time: *r*=–.48, *p*<.001; accuracy: *r*=–.57, *p*<.001). That is, greater change in affective control was associated with fewer mental health problems. In contrast, the Placebo group showed small, non-significant associations between change in affective control and post-training mental health difficulties, (reaction time: *r*=.21, *p*=.065, accuracy: *r*=–.28, *p*=.014). However, the effects of training on mental health difficulties were not maintained at the one-month follow-up (reaction time: *X_Diff_^2^*=4.54, *p*=.10, accuracy: *X_Diff_^2^*=2.43, *p*=.30, nor at one-year follow-up (reaction time: *X_Diff_^2^*=0.87, *p*=.65, accuracy: *X_Diff_^2^*=0.89, *p*=.64).

There was no effect of AffeCT compared to Placebo on emotion regulation difficulties or self-control capacity. This was true for latent growth curve models including the latent affective control index for reaction time (emotion regulation: *X_Diff_^2^*=3.61, *p*=.163; self-control: *X_Diff_^2^*=3.67, *p*=.163) and accuracy (emotion regulation: *X_Diff_^2^*=0.00, *p*=.999; self-control: *X_Diff_^2^*=4.98, *p*=.081).

## Discussion

Identifying novel and scalable avenues for prevention of mental problems is essential (Hagan et al., 2015), as mental ill health has become the leading burden of disease in young people worldwide (Gore et al., 2011). The present study tested the potential of an app-based affective control training to benefit adolescents’ mental health. The study found that adolescents’ performance on an affective control training task improved from pre- to post-training, with older adolescents benefiting more from training compared to younger adolescents. These training gains did not lead to improvements on non-trained measures of affective control, across affective updating, inhibition and shifting. However, variance of change in affective control was related to reduced mental health difficulties at pre-training in the AffeCT but not Placebo group. This training-related benefit was not maintained one-month or one-year later. We discuss the implications of these findings for the potential of app-based affective control training in mental health.

### Improving affective control with minimal effort

The observed improvement on the trained AffeCT task, was noteworthy as the AffeCT group on average completed half as many training sessions as the Placebo group. Moreover, both groups predominantly completed the “simpler” training tasks. For the AffeCT group this were the two single modality training versions. That is, adolescents limited the cognitive effort involved in training by selecting the less demanding option. Ganesan & Steinbeis (2021) argued that effort exerted in cognitive (training) tasks is guided by an individual’s cost-value computation. This suggests that the perceived value of training, especially on the dual *n*-back version of AffeCT, was insufficient to motivate most participants. Engagement in cognitive training tasks, such as AffeCT, should therefore be motivated by providing appropriate incentives to exert cognitive effort. The incentives are not limited to monetary incentives or other rewards, instead the relative value of “training” can be increased by changing adolescents’ mindset about the benefits of a specific training regime (Yeager et al., 2022).

### Age-related training improvement

Pre- to post-training gains in affective control (i.e., change in maximum level of *n* back achieved from pre- to post-training) increased as a function of age. This is in line with non-affective cognitive training findings, which have shown greater benefits of cognitive training in older (16-18 years) compared to younger (11-13 years) adolescents (Knoll et al., 2016). A comprehensive review of the literature on brain plasticity in adolescence, however, suggests that brain plasticity-related changes in higher cognitive functions during adolescence are dependent on the brain regions involved in the specific cognitive domain that is being trained and a range of factors, including the type of training and the trained individuals’ gender (Laube, van den Bos, & Fandakova, 2020). Affective control recruits the cognitive control network, in particular the inferior frontal gyrus (S. Schweizer, Satpute, et al., 2019), which shows protracted development throughout adolescence. The age-related differences in training gains observed in the present study support training-induced functional changes and possibly structural plasticity of the neural substrates of affective control throughout adolescence. However, examinations of training-induced functional or structural brain changes in typically developing adolescents are scarce (Lee, Kwak, & Chey, 2019). Examining training-induced neural changes will be important to determine potentially sensitive periods for training (Frankenhuis & Walasek, 2020).

### Performance on different facets of affective control remains unchanged by AffeCT

AffeCT did not transfer to significantly greater improvements in affective inhibition (Preston & Stansfield, 2008), affective working memory (S. Schweizer, Leung, et al., 2019) and affective shifting (S. Schweizer, Parker, et al., 2020). The lack of training-related transfer to untrained measures of affective control is in line with meta-analytic reviews of the cognitive control training literature, which show that training leads to improvements on the trained task but typically does not extend to untrained measures of neutral cognitive control (e.g., Soveri et al., 2017). Transfer effects (i.e., training-induced changes) to *affective* control have received comparatively less attention. In older adolescents (16-24 years), Roberts et al. (2021) showed that neutral, but not affective control training, led to improvements in affective updating. In adults, cognitive (Cohen et al., 2016; Sari, Koster, Pourtois, & Derakshan, 2016) and affective (S. Schweizer et al., 2013; S. Schweizer, Hampshire, & Dalgleish, 2011) control training paradigms have been shown to improve affective inhibition. While the current study showed no transfer on untrained affective control measures, the existent literature suggests that with appropriate training regimes facets of affective control may be malleable and benefit from computerized control training.

### Affective control gains and mental health

Despite the lack of training-related transfer to the individual facets of affective control, pre- to post-training change on a composite index of affective control and pre- to post-training change in mental health problems covaried in the AffeCT group but not the Placebo. That is, greater improvement in affective control was associated with mental health benefits in adolescents who had trained with AffeCT but not Placebo. This is in line with a review of the literature showing that computerized cognitive control training, in particular training that induces improvements in affective control, is associated with benefits in symptoms of depression and anxiety in children and adolescents (Edwards et al., 2022) and adults (Koster, Hoorelbeke, Onraedt, Owens, & Derakshan, 2017).

Of note is the per protocol use of multi-group latent growth curve modelling to analyse the impact of training. Traditional methods to analyse training effects have typically included repeated measures analyses of variance and mixed effects models that examine average performance or symptom levels (Soveri et al., 2017). Multi-group latent growth curve modelling instead compared the groups on the extent to which individual variance in the change in affective control was associated with variance in change in mental health benefits. This training effect would not have been captured by traditional inference methods and despite not extending beyond the immediate post-training assessment, it warrants future investigation into the potential of affective control training as a preventative intervention in adolescent mental health. A particularly promising avenue of investigation is whether the training benefits of AffeCT can be augmented in magnitude (i.e., more reduction in symptoms) and temporally extended when increasing engagement and participation in the more demanding version of the training task (i.e., dual vs. single *n*-back). Beyond increasing engagement by an improved rationale for training as proposed above, participation can be boosted through gamification. Gamification refers to the process of enhancing training with affordances to create engaging experiences (Hamari, 2013). Meta-analytic evidence demonstrates that it is an effective tool to reliably encourage attentional engagement and motivation to increase rates of training (Lumsden, Edwards, Lawrence, Coyle, & Munafò, 2016).

The present findings need to be considered within the context of the study’s limitations. While the study was adequately powered, limited per protocol engagement with the task restricts the inferences that can be drawn regarding the effectiveness of affective control training. Moreover, for over half the sample the one-year follow-up was conducted during the COVID-19 pandemic, so the potential to detect training-effects over and above the adverse impact that the pandemic has had on adolescent mental health (Racine et al., 2021) is limited. Moreover, the assessment of mental health in the current study arguably does not capture potential effects of affective control training on affective fluctuations in adolescents’ everyday lives. Future research should therefore consider assessing the impact of AffeCT on dynamically changing mood states by sampling affect using ecological momentary assessments.

In sum, the present study showed that affective control training in adolescents, especially older adolescents, led to improvements in performance on the training task, but did not transfer to untrained measures of individual facets of affective control. Encouragingly, the covariance of change on the non-trained composite index of affective control and mental health difficulties form pre- to post-training showed a significant benefit of AffeCT over Placebo. That is, the present study provides preliminary evidence that affective control training may confer short-term preventative benefits for adolescent mental health. App-based training is easy to disseminate and can therefore be delivered at scale anywhere in the world. Even small and short-term benefits are therefore potentially meaningful if they can be delivered at the population level. If engagement with affective control training can be further boosted through gamification and incentives, these benefits may be extended beyond the period immediately following training.

## Supplementary Material

Supplementary Material

## Figures and Tables

**Table 1:** Baseline Characteristics Across Groups

	Placebo		AffeCT			
	*M / N*	*SD / %*	*M / N*	*SD / %*	*t/X^2^*	*p*
Age	14.32	*2.35*	14.33	2.28	–0.03	.975
Gender					1.41	.495
Female	79	81	80	78		
Male	16	16	21	21		
Other	3	3	1	1		
Parental education (SES proxy)					6.31	.788
General secondary education	9	9	8	8		
Advanced secondary education	14	14	13	13		
Undergraduate degree	19	20	19	18		
Postgraduate degree	18	18	21	21		
Missing	38	39	41	40		
Ethnicity					1.43	.699
Asian	19	20	23	22		
Black	13	13	18	18		
White	53	54	49	48		
Mixed/Other	13	13	12	12		
Fluid intelligence	8.34	2.13	7.85	2.25	1.51	.132
Mental health difficulties	14.89	3.80	16.08	4.72	–1.93	.055
Emotion regulation difficulties	46.85	15.97	50.21	20.83	–1.27	.204
Self-control	23.82	6.52	23.04	7.25	0.79	.430
Group size	21.94	13.38	21.88	12.87	0.03	.975

*Note*. The table reports baseline characteristics and demonstrates that randomisation was successful with Bayesian *t*-tests (continuous variables) and *Chi* square tests (categorical variables) showing no significant group differences on any baseline characteristics. Fluid intelligence = IQ score derived from the Raven’s Advanced Progressive Matrices (Raven, Court, & Raven, 1988); Mental health difficulties = Difficulties score on the Strengths and Difficulties Questionnaire (Goodman, 1997); Emotion regulation difficulties = Total score on the Difficulties in Emotion Regulation Scale (Gratz & Roemer, 2004); Self-control = Total score on the Brief Self-control Scale (Tangney, Baumeister, & Boone, 2004); Parental education = Highest parental education was measured as a proxy of socioeconomic status (SES); Asian = Included individuals selecting any of these answer options: Asian-other, Bangladeshi, Chinese, Indian, Pakistani; Black = Black is a term used in Britain to refer to citizens of African or African-Caribbean decent, here it included individuals who selected any of the following to describe their ethnicity: Black-African, Black-British, Black-Other; White = here refers to individuals who identified as White-British or White-other; Mixed/Other = here includes individuals who identified as being of mixed or other ethnicity than the available options by selecting Mixed/Other; Group size = average number of participants in the pre- and post-training assessment sessions.

**Table 2:** Mixed Effects Models Investigating the Effects of Training Group on Affective Control Facets from Pre- to Post-Training

	Accuracy				Reaction time			
	*B*	*SE*	*t*	*p*	*B*	*SE*	*t*	*p*
Affective inhibition								
Intercept	–0.01	0.01	–1.46	.143	–439.90	8.76	–50.22	<.001
Time	0.00	0.01	0.25	.810	–622.14	12.38	–50.24	<.001
Group	–0.00	0.01	0.05	.957	19.80	12.46	1.59	.112
Time × Group	0.00	0.01	0.46	.646	28.02	17.62	1.59	.112
Affective shifting								
Intercept	0.00	0.02	0.19	.850	–831.11	40.33	–20.61	<.001
Time	–0.03	0.03	–1.07	.287	–1176.22	56.97	–20.65	<.001
Group	–0.00	0.03	–0.13	.895	44.54	57.60	0.77	.439
Time × Group	0.01	0.04	0.26	.796	62.59	81.36	0.77	.442
Affective updating								
Intercept	–0.19	0.14	–1.37	.170				
Time	–0.04	0.12	–0.33	.745				
Group	–0.04	0.20	–0.21	.838				
Time × Group	–0.01	0.17	–0.06	.955				

*Note*. Time=Pre-training vs. Post-training; Group=AffeCT vs. Placebo. The models are reported for accuracy and reaction time performance on affective inhibition (affective Stroop task), shifting (affective card sorting task) and updating task (affective backward digit span task). For means and standard deviations across the different conditions at pre- and post-training on the affective inhibition, shifting and updating tasks see Table S3.
