# Supplementary material for: Changes in Affective Control Covary with Changes in Mental Health Difficulties Following Affective Control Training (AffeCT) in Adolescents
Source: Psychol Med. Author manuscript; Available in PMC 2024 Feb 29. (PMC7615678; doi:10.1017/S0033291723002167)
Supplement: Supplementary Material [file EMS190286-supplement-Supplementary_Material.docx]

**Supplementary Methods**

**Figure 1**

*Participant flow chart*

**Training task progression**

The first three days of the training started at n = 1, and from day 4 onwards the training started at the average level achieved in the previous training session. Difficulty level increased across training blocks within a session if performance accuracy reached 70% and above, and it decreased if performance accuracy was 30% or below. Feedback was provided after each response, with a red border around the grid for false alarms (pressing “Match” on non-target trials) or misses (pressing “No match” on target trials or no response), and a green border for correct trials.

**Supplementary Results**

**Training adherence**

The groups did not significantly differ in the total mins trained, *t* (90) = 0.93, *p* = .350. Individuals in the Placebo group trained for 17.63 mins on average (*SD* = 32.3), whereas the AffeCT group trained for 12.32 mins on average (*SD =*20.27). The groups did not significantly differ in the ratio of time spent on the more challenging and as per the rationale provided to participants more beneficial C version of the training task versus the A and B versions of the training tasks, *t* (90) = 0.50, *p* = .618. The ratio was controlled for overall minutes trained (i.e. ((time spent on C – time spent on A+B)/total time spent training)). In the case of the Placebo group the C version of the task was not actually more challenging or beneficial than the A and B versions.

**Figure S1**

**A**

**
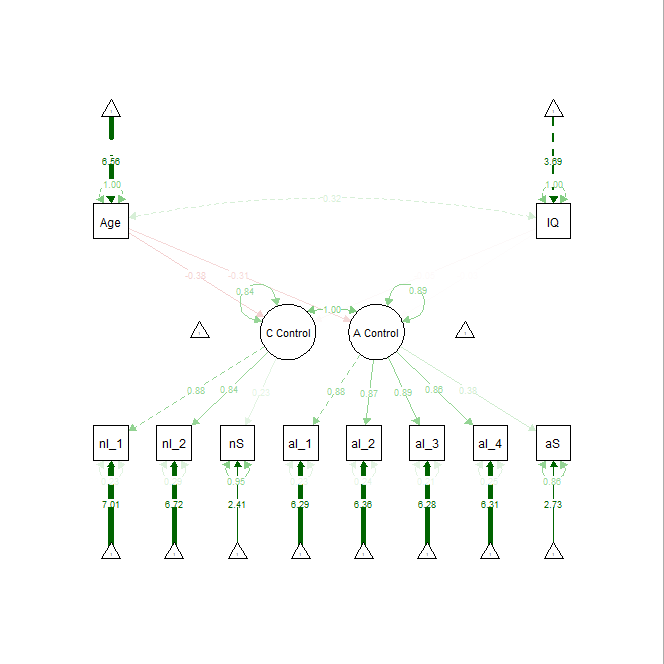
**

**B**

**
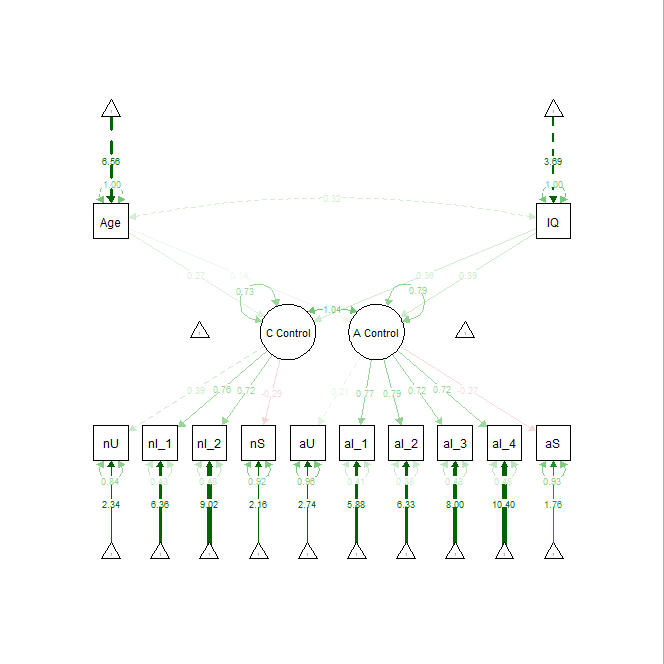
**

*Note.* Panel A shows the structure of the reaction time data and panel B shows the accuracy data. nI_1 = Neutral Inhibition 1, reflecting reaction time/accuracy on neutral Stroop trials for sad words; nI_2 = Neutral Inhibition 2, reflecting reaction time/accuracy on neutral Stroop trials for happy words; nS = Neutral Shifting, reflecting reaction time/accuracy on card sorting trials in the shape condition; nU = Neutral Updating, reflecting maximum digit span achieved in the neutral condition; aI_1 = Affective Inhibition 1, reflecting reaction time/accuracy on incongruent Stroop trials for sad words; aI_2 = Affective Inhibition 2, reflecting reaction time/accuracy on incongruent Stroop trials for happy words; aI_3 = Affective Inhibition 3, reflecting reaction time/accuracy on congruent Stroop trials for sad words; aI_4 = Affective Inhibition 4, reflecting reaction time/accuracy on congruent Stroop trials for happy words; aS = Affective Shifting, reflecting reaction time/accuracy on card sorting trials in the emotional expression condition; aU = Affective Updating, reflecting maximum digit span achieved in the affective condition.

**Table S1**

*Fit Indices of Two-Factor (Affective and Cognitive Control) vs. One-Factor (Cognitive Control) Models*

|  | *Χ^2^* | *Df* | CFI | TLI | RMSEA | SRMR | AIC |
| --- | --- | --- | --- | --- | --- | --- | --- |
| Reaction time |  |  |  |  |  |  |  |
| Two-factor model | 48.84 | 31 | .98 | .97 | .06 | .05 | –176.18 |
| One-factor model | 53.11 | 34 | .98 | .97 | .06 | .06 | –177.10 |
| Accuracy |  |  |  |  |  |  |  |
| Two-factor model | 100.75 | 31 | .81 | .73 | .11 | .10 | –434.56 |
| One-factor model | 133.50 | 34 | .74 | .66 | .14 | .11 | –403.07 |

*Note.* The table reports the model fit indices for the model structure of task performance on the affective Stroop (Preston & Stansfield, 2008), affective card sorting task (Schweizer, Parker, et al., 2020) and affective backward digit span task (Schweizer, Leung, et al., 2019). Specifically, the models compared the predicted two-factor structure (i.e., separate affective control and cognitive control factors) to the more parsimonious one-factor structure (general cognitive control factor). For reaction time the two-factor model included the following observed variables in the affective control factor: sad and happy incongruent and congruent Stroop trials, affective card sorting condition. The cognitive control factor included: neutral Stroop trials and the neutral version of the card sorting task. For accuracy, the two-factor model included the following observed variables in the affective control factor: affective backward digit span level, sad and happy incongruent and congruent Stroop trials, affective card sorting condition. The cognitive control factor included: neutral backward digit span level neutral Stroop trials and the neutral version of the card sorting task. CFI = comparative fit index; TLI = Tucker-Lewis Index; RMSEA = root mean square error of approximation; SRMR = standardized root mean square residual; AIC = Akaike Information Criterion.

**Table S2**

*Effects of Time and Training Group on Performance on the Training Task*


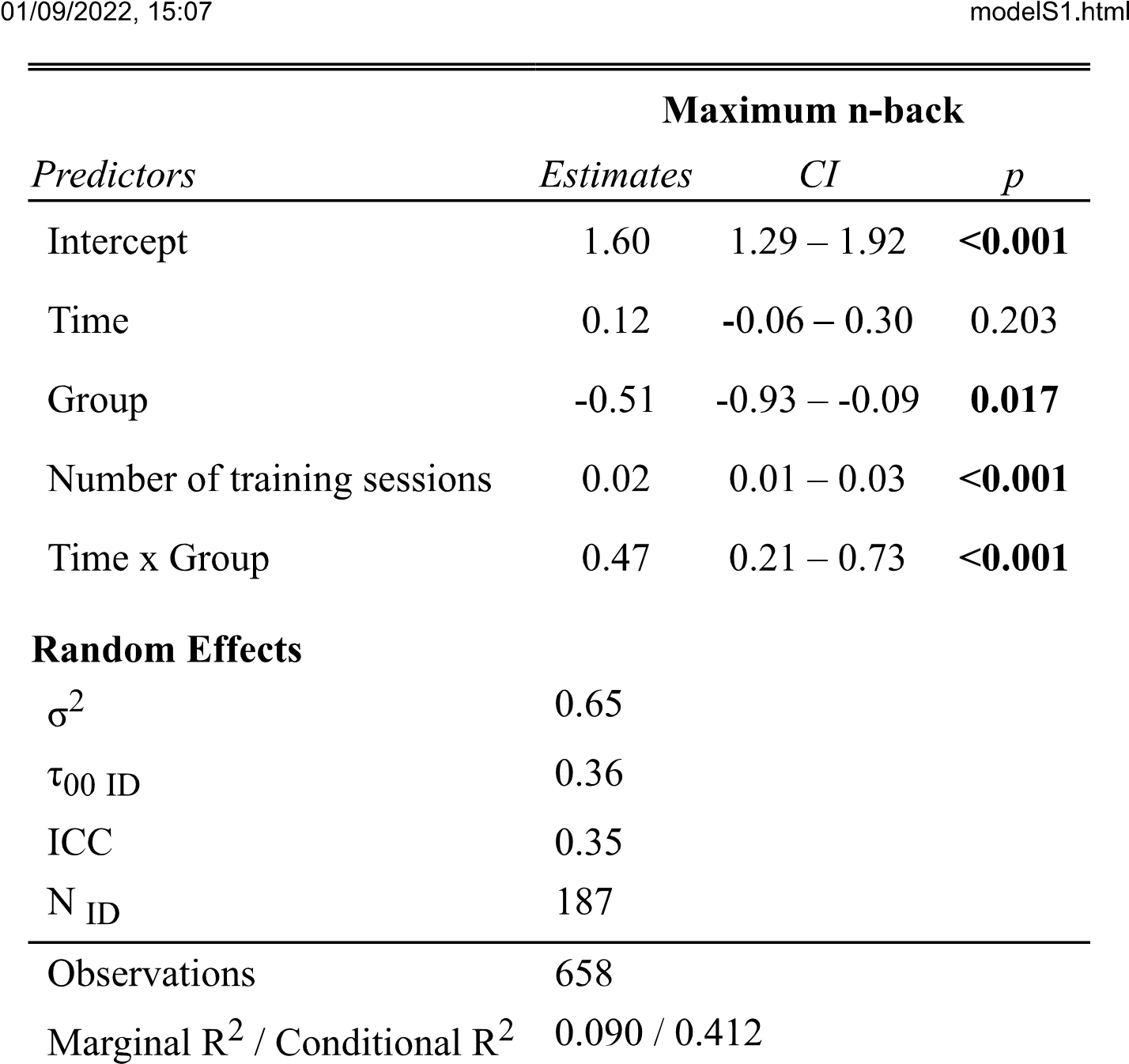


*Note.* Time = Pre-training vs. post-training; Group = AffeCT vs. Placebo; Number of training sessions = number of sessions completed that were 10 minutes or longer. Blod print indicates a significant result, *p* < .05. Marginal *R^2^* = .09; Conditional *R^2^* = .41.

**Table S3**

*Means and standard deviations across different conditions at pre- and post-training on the affective inhibition, shifting, and updating tasks.*

|  | Pre-training | | | | | | | Post-training | | | | | | | |
| --- | --- | --- | --- | --- | --- | --- | --- | --- | --- | --- | --- | --- | --- | --- | --- |
|  | *AffeCT* | | | *Placebo* | | | | *AffeCT* | | | | *Placebo* | | | |
|  | *Accuracy*  *M (SD)* | | *RT*  *M (SD)* | | *Accuracy*  *M (SD)* | | *RT*  *M (SD)* | | *Accuracy*  *M (SD)* | *RT*  *M (SD)* | | | *Accuracy*  *M (SD)* | | *RT*  *M (SD)* |
| Affective inhibition |  |  | |  | |  | |  | | |  |  | |  | |
| Happy | -0.02 (0.11) | -0.91 (0.19) | | 0 (0.07) | | -0.93 (0.22) | | -0.02 (0.07) | | | -0.84 (0.19) | 0 (0.06) | | -0.86 (0.18) | |
| Sad | -0.01 (0.09) | -0.93 (0.20) | | -0.02 (0.09) | | -0.96 (0.21) | | -0.01 (0.09) | | | -0.85 (0.19) | 0.01 (0.11) | | -0.90 (0.19) | |
| Affective shifting |  |  | |  | |  | |  | | |  |  | |  | |
| Affective | 0.25 (0.22) | 2.94 (1.59) | | 0.27 (0.25) | | 2.94 (1.49) | | 0.20 (0.21) | | | 1.69 (0.91) | 0.20 (0.20) | | 1.97 (1.02) | |
| Neutral | 0.24 (0.19) | 2.14 (0.92) | | 0.25 (0.21) | | 2.28 (1.48) | | 0.23 (0.21) | | | 1.61 (0.79) | 0.21 (0.21) | | 1.65(0.66) | |
| Affective updating |  |  | |  | |  | |  | | |  |  | |  | |
| Affective | 5.28 (1.38) | -- | | 5.32 (1.79) | | -- | | 5.15 (1.69) | | | -- | 5.59 (1.95) | | -- | |
| Neutral | 5.37 (1.49) | -- | | 5.47 (1.51) | | -- | | 5.42 (1.66) | | | -- | 5.84 91.78) | | -- | |

*Note.* Affective inhibition was measured as incongruency index (accuracy/reaction time on incongruent trails - accuracy/reaction time on neutral trails) on the Stroop task, which requires adjectives to be categorised as happy or sad over incongruent background faces (affective condition) or scrambled background faces (neutral condition) (Preston & Stansfield, 2008). Affective shifting was indexed by subtracting the number of errors made/reaction time in the colour and number trials of the neutral condition (i.e., condition including shapes) from the number of errors made/reaction time in the affective condition (i.e., condition including faces with emotional expressions). Affective updating was operationalised by subtracting the backward digit span length in the neutral condition (i.e., digits presented over neutral background images) from the span length affective in the affective condition (i.e., digits presented over affective background images).

**Table S4**

*Effects of Time, Training Group and Age on Performance on the Training Task*


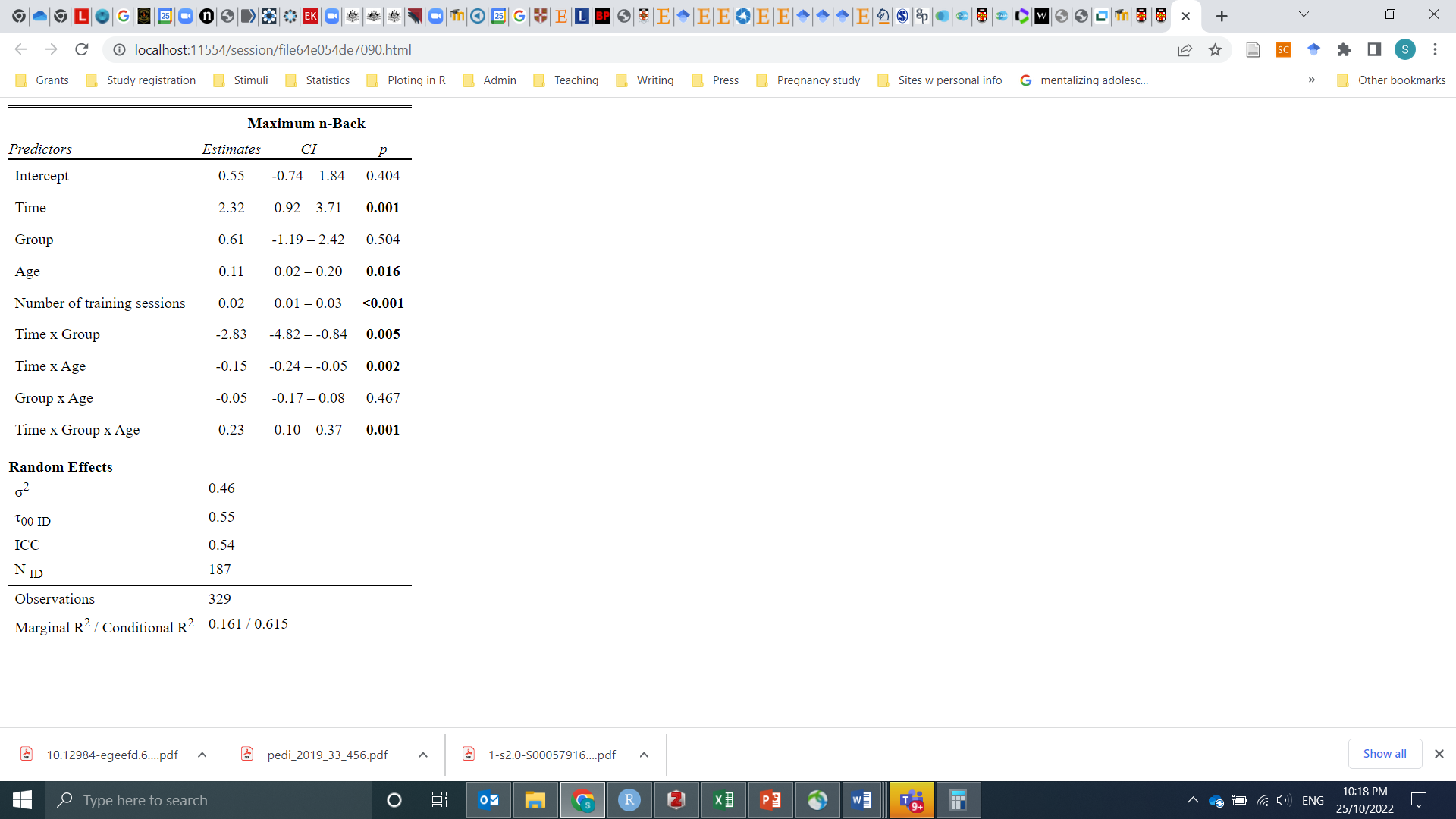


*Note.* Time = Pre-training vs. post-training; Group = AffeCT vs. Placebo; Number of training sessions = number of sessions completed that were 10 minutes or longer. Blod print indicates a significant result, *p* < .05. Marginal *R^2^* = .16; Conditional *R^2^* = .62.

**Table S5**

*Multi-Group (AffeCT vs. Placebo) Latent Growth Curve Models of Affective Control and Mental Health Difficulties from Pre- to Post-Training*

|  | *Χ^2^* | *Df* | CFI | TLI | RMSEA | SRMR | AIC | *β* | *SE* | *z* | *p* |
| --- | --- | --- | --- | --- | --- | --- | --- | --- | --- | --- | --- |
| **Reaction Time** | | | | | | | | | | | |
| Free model fit |  |  | 1.00 | 1.00 | .02 | .09 | 1221.23 |  |  |  |  |
| Constrained model fit |  |  | .99 | .99 | .03 | .10 | 1225.97 |  |  |  |  |
|  | Placebo Group | | | | | | | | | | |
| Regressions |  | | | | | | | | | | |
| ΔMental health  ~Affective control pre-training |  |  |  |  |  |  |  | −0.02 | 1.94 | −0.01 | .99 |
| ~Mental health pre-training |  |  |  |  |  |  |  | −0.31 | 0.09 | −3.56 | <.001 |
| ΔAffective control  ~Mental health pre-training |  |  |  |  |  |  |  | 0.01 | 0.01 | 2.30 | .02 |
| ~Affective control pre-training |  |  |  |  |  |  |  | −0.36 | 0.09 | −4.17 | <.001 |
| Covariance  Affective control pre-training ~~ Mental health pre-training |  |  |  |  |  |  |  | 0.13 | 0.07 | 1.74 | .08 |
| ΔAffective control ~~ ΔMental health |  |  |  |  |  |  |  | 0.03 | 0.04 | 0.61 | .54 |
|  | AffeCT Group | | | | | | | | | | |
| Regressions |  | | | | | | | | | | |
| ΔMental health  ~Affective control pre-training |  |  |  |  |  |  |  | −1.68 | 1.91 | −0.88 | .38 |
| ~Mental health pre-training |  |  |  |  |  |  |  | −0.17 | 0.11 | −1.50 | .14 |
| ΔAffective control  ~Mental health pre-training |  |  |  |  |  |  |  | −0.01 | 0.00 | −1.99 | .05 |
| ~Affective control pre-training |  |  |  |  |  |  |  | −0.33 | 0.09 | −2.04 | .04 |
| Covariance |  |  |  |  |  |  |  |  |  |  |  |
| Affective control pre-training ~~ Mental health pre-training |  |  |  |  |  |  |  | 0.02 | 0.10 | 0.23 | .82 |
| ΔAffective control ~~ ΔMental health |  |  |  |  |  |  |  | −0.19 | 0.09 | −2.04 | .04 |
| **Accuracy** | | | | | | | | | | | |
| Free model fit |  |  | .69 | .66 | .14 | .19 | −115.19 |  |  |  |  |
| Constrained model fit |  |  | .69 | .66 | .14 | .19 | −109.43 |  |  |  |  |
|  | Placebo Group | | | | | | | | | | |
| Regressions |  | | | | | | | | | | |
| ΔMental health  ~Affective control pre-training |  |  |  |  |  |  |  | −0.88 | 1.48 | −0.59 | .55 |
| ~Mental health pre-training |  |  |  |  |  |  |  | −0.30 | 0.09 | −3.35 | .001 |
| ΔAffective control  ~Mental health pre-training |  |  |  |  |  |  |  | −0.03 | 0.01 | −2.87 | .004 |
| ~Affective control pre-training |  |  |  |  |  |  |  | −0.17 | 0.17 | −0.98 | .33 |
| Covariance |  |  |  |  |  |  |  |  |  |  |  |
| Affective control pre-training ~~ Mental health pre-training |  |  |  |  |  |  |  | 0.14 | 0.13 | 1.15 | .25 |
| ΔAffective control ~~ ΔMental health |  |  |  |  |  |  |  | 0.02 | 0.12 | 0.18 | .86 |
|  | AffeCT Group | | | | | | | | | | |
| ΔMental health  ~Affective control pre-training |  |  |  |  |  |  |  | −0.05 | 1.18 | −0.04 | .97 |
| ~Mental health pre-training |  |  |  |  |  |  |  | −1.19 | 0.10 | −1.98 | .05 |
| ΔAffective control  ~Mental health pre-training |  |  |  |  |  |  |  | −0.01 | 0.01 | −0.69 | .49 |
| ~Affective control pre-training |  |  |  |  |  |  |  | −0.19 | 0.14 | −1.46 | .17 |
| Covariance |  |  |  |  |  |  |  |  |  |  |  |
| Affective control pre-training ~~ Mental health pre-training |  |  |  |  |  |  |  | −0.08 | 0.24 | −0.32 | .75 |
| ΔAffective control ~~ ΔMental health |  |  |  |  |  |  |  | −0.67 | 0.21 | −3.13 | .002 |

*Note.* CFI = comparative fit index; TLI = Tucker-Lewis Index; RMSEA = root mean square error of approximation; SRMR = standardized root mean square residual; AIC = Akaike Information Criterion.
